# “I’M INDEPENDENT. SO FAR...”: INVESTIGATING INFORMAL CAREGIVING PATTERNS OF OLDER ADULTS WITH PRIMARY LIVER CANCER

**DOI:** 10.1093/geroni/igad104.2972

**Published:** 2023-12-21

**Authors:** Deborah Watman, Sasha Perez, Yingtong Chen, Sara Lubetsky, Christopher Woodrell

**Affiliations:** Icahn School of Medicine at Mount Sinai, New York City, New York, United States; Icahn School of Medicine at Mount Sinai, New York City, New York, United States; Icahn School of Medicine at Mount Sinai, New York City, New York, United States; Icahn School of Medicine at Mount Sinai, New York City, New York, United States; Icahn School of Medicine at Mount Sinai, New York City, New York, United States

## Abstract

Older adults with advanced stage hepatocellular carcinoma (HCC) are living longer given improved treatment effectiveness. Little is known about their unmet supportive care needs and their caregivers’ needs, some of which may be addressed by palliative care. Previous studies of palliative care in populations facing liver disease have cited difficulty with recruitment. This is a cross-sectional study at a single academic medical center in NYC that uses validated survey instruments and qualitative interviews to explore caregiver support for those receiving treatments for HCC. We recruited patients with liver disease, and caregivers of patients 60 years or older. Validated survey instruments were used to collect data and a qualitative interview is conducted that focuses on the experience of supporting a loved one with HCC. 65% (13/20) participants were over the age of 60. PDSA framework was used to improve recruitment to four caregivers thus far. Results showed that language matters. Recruitment materials were updated from “palliative” to “supportive” care, to reduce stigma and fear of worsening health status and the perception of burden. Older adults value their independence and are not likely to identify a “caregiver”. They may be more willing to identify a loved one who is there to help. 90% of older adults identified a caregiver using the Duke Social Support Index. Thoughtfulness and adaptability are necessary when recruiting amongst this vulnerable yet increasingly independent population. Furthermore, older adults facing liver disease may be solo agers with no caregiver, increasing the need for supportive care as their disease progresses.

